# Mechanical Stress Stimulates the Osteo/Odontoblastic Differentiation of Human Stem Cells from Apical Papilla via ERK 1/2 and JNK MAPK Pathways

**DOI:** 10.1155/2014/494378

**Published:** 2014-04-15

**Authors:** Chao Mu, Taohong Lv, Zilu Wang, Shu Ma, Jie Ma, Jin Liu, Jinhua Yu, Jinquan Mu

**Affiliations:** ^1^State Key Laboratory of Reproductive Medicine, Department of Stomatology, Nanjing Maternity and Child Health Care Hospital Affiliated to Nanjing Medical University, Nanjing, Jiangsu 210029, China; ^2^Institute of Stomatology, School of Stomatology, Nanjing Medical University, 136 Hanzhong Road, Nanjing, Jiangsu 210029, China; ^3^Endodontic Department, School of Stomatology, Nanjing Medical University, 136 Hanzhong Road, Nanjing, Jiangsu 210029, China

## Abstract

*Background Information*. Stem cells from apical papilla (SCAPs) are a potent candidate for the apexogenesis/apexification due to their multiple differentiation capacity. During the orthodontic treatment of developing teeth, SCAPs *in vivo* are usually subjected to the cyclic stress induced by compression forces. However, it remains unclear whether mechanical stress can affect the proliferation and differentiation of human SCAPs. *Results*. Human SCAPs were isolated and stimulated by 200 g mechanical stimuli for 30 min and their proliferation and differentiation capacity were evaluated *in vitro* at different time points. MTT and FCM results demonstrated that cell proliferation was enhanced, while TEM findings showed the morphological and ultrastructural changes in stress-treated SCAPs. ALP activity and mineralization capacity of stress-treated SCAPs were upregulated . In the meantime, higher odontogenic and osteogenic differentiation were found in stress-treated SCAPs by real-time RT-PCR and Western blot, as indicated by the expression of related markers at both mRNA and protein levels. Moreover, the protein expressions of pJNK and pERK MAPK pathways were upregulated. *Conclusion*. Together, these findings suggest that mechanical stress is an important factor affecting the proliferation and differentiation of SCAPs via the activation of ERK and JNK signaling pathway.

## 1. Introduction

Human stem cells from apical papilla (SCAPs) are considered to be potential candidates for bone/tooth tissue engineering because they are uncommitted and multipotent cells with the ability to become specialized cells and they can be easily isolated from the root apex of immature permanent teeth [1, 2]. Many clinical trials have proved that the apexogenesis of immature tooth and root development in teenagers suffering from the endodontic diseases can undergo the spontaneous repair through the continuous formation of root primarily contributed by SCAPs [3]. In the meantime, teeth are load-bearing structures; and they are capable of sensing mechanical stimuli in their local environment, interpreting these stimuli, and responding in a biologically appropriate fashion in the routine mastication [4–6]. During the orthodontic treatment of immature permanent teeth, apical structures will remodel themselves to coincide with the orthodontic forces, in which SCAPs may play a paramount role in the root maturation and apexogenesis. However, the impact of these biomechanical factors on the biological features of SCAPs has yet to be fully elucidated.

Clinically, mechanical stress is known to be one of the important factors in shaping the roots of teeth in orthodontics and plays a significant role in the regulation of bone remodeling during orthodontic tooth movement [7]. Besides, previous studies have shown that the application of mechanical loading on cultured mesenchymal stem cells (MSCs) may trigger their osteogenic differentiation with or without the addition of growth factors [8–10]. The role of mechanical stimulation in the regulation of the fate of MSCs is of interest in bone regeneration and tissue engineering [11–13]. To data, whether orthodontic forces can affect the differentiation of human SCAPs remains unclear. Furthermore, the signaling mechanisms involved in the stress-induced regulation of cell differentiation remain to be completely defined [14].

To gain a better understanding of the role of mechanical loads in the root development and differentiation of SCAPs, we used mechanical stress to simulate the orthodontic force and to investigate the stress-induced differentiation of SCAPs as well as the mechanism of mechanotransduction associated with the MAPK pathway. Our findings demonstrated that the odonto/osteogenic differentiation potential of SCAPs was enhanced in which ERK 1/2 and JNK were upregulated, indicating that the mechanical stress may regulate the differentiation of SCAPs via MAPK pathway. These findings also contribute to a better understanding of stress-induced bone/tooth remodeling.

## 2. Materials and Methods

### 2.1. Culture of Human SCAPs

Primary human SCAPs derived from impacted third molars were collected from patients (17–20 years old, *n* = 36) at the Department of Stomatology in Nanjing Maternity and Child Health Care Hospital after informed consents were obtained. The apical papillae were gently separated from the root surface and then digested in a solution of 3 mg/mL collagenase type I (Sigma-Aldrich Chemie, Taufkirchen, Germany) and 4 mg/mL dispase (Roche, Mannheim, Germany) for 30 min at 37°C. These cells were characterized to the standard procedures for magnetic activated cell sorting (MACS) by using rabbit anti-STRO-1 antibody (Santa Cruz, Delaware, CA) and sheep anti-rabbit IgG Dynabeads (Dynal Biotech, Oslo, Norway). Cells were cultured in *α*-minimum essential medium (*α*-MEM, Gibco, Life Technologies, Grand Island, NY) supplemented with 10% fetal bovine serum (FBS, Gibco), 100 *μ*mol/L ascorbic acid 2-phosphate (Sigma), 2 mmol/L glutamine (Gibco), 100 U/mL penicillin, and 100 *μ*g/mL streptomycin at 37°C in 5% CO_2_. Culture media were replaced every 3 days. Cells were subcultured when they reached 80–90% confluence. SCAPs at passage 2-3 were used for the subsequent experiments. Cells were seeded and allowed to adhere for 2-3 h. Then cells were cultured in supplemented media for 72 h, and serum-starved for another 24 h for cell synchronization, after which cells were exposed to mechanical stress.

### 2.2. Mechanical Stress Application

**Figure 1:**
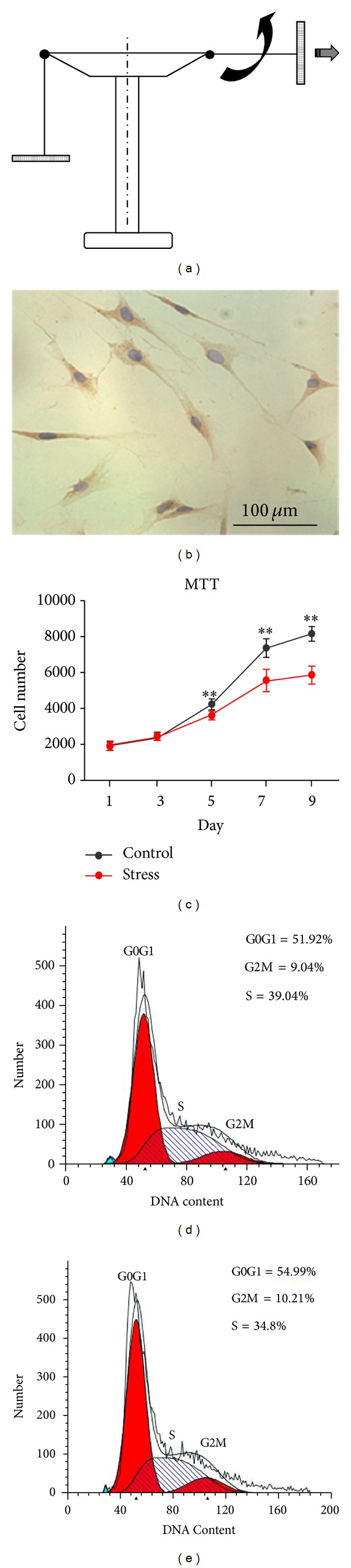
Schematic diagram of the experimental setup (side view), immunocytochemical staining of STRO-1, and effects of mechanical stress on the proliferation of SCAPs* in vitro*. (a) Left: the bucket is in resting position; right: the bucket is swung out because of centrifugal force. The thick arrows indicate the direction of force. (b) Immunocytochemical staining for STRO-1 in SCAPs. (c) Growth curves of mechanical stress-treated SCAPs. Values are described as the means ± SD, *n* = 3. (d), (e) Flow cytometry analysis for mechanical stress-untreated (d) and treated (e) SCAPs.

SCAPs were plated onto six-well plates (Costar, USA) and cultured at 37°C in a CO_2_ incubator. When cells reached 70% confluence, centrifugal force was applied by a centrifuge (Allegra 64R, Beckman Coulter, USA) equipped with specially designed rotors to hold six-well or ninety six-well culture plates. The strain experienced by the cells in the culture plates was provided through a top-bottom axis (Figure 1(a)). Actually the force (*F*) is decided by the centrifugal radius of the centrifuge (*R*, in cm) and rotates speed (RS, revolutions per min). Consider *F* = 1.18 × 10^−5^ × *R* × RS^2^ (Figure 1(a)). Then SCAPs were cultured in the plates and centrifuged at 200 g (corresponded to a speed at 1000 rpm) for 30 min [15], which resembles the clinical orthodontic force, and then returned to the incubator. Control cells were cultured in absence of centrifugal loading. Proliferation and differentiation of SCAPs were assessed at different time points.

### 2.3. Cell Morphology and Ultrastructure

Morphological changes of untreated and treated SCAPs were evaluated at day 7 by a computer-control digit imaging system (Olympus, Japan). Meanwhile, ultrastructural analysis was carried out with 1 × 10^7^ cells which were collected by centrifugation to deposit the cell pellets. The pellets were fixed in a 2.5% glutaraldehyde solution at 4°C overnight and then examined by transmission electron microscopy (TEM, JEM-2000EX, Japan).

### 2.4. MTT Assay and Cell Growth Curve

MTT (Sigma-Aldrich, St. Louis, MO, USA) assay was used to evaluate the proliferation of SCAPs based on quantitating the insoluble purple formazan through colorimetric assay, which comes from mitochondrial succinic dehydrogenase. In brief, cells were stimulated after 24 h serum-starved and then cultured in 96-well plates (Costar, Cambridge, MA) in *α*-MEM containing 10% FBS at the density of 5 × 10^3^ cells/well. 20 *μ*L fresh MTT solution (5 mg/mL) was added into the wells and incubated at 37°C for 4 h in the next 7 consecutive days. Cells were washed twice with 0.01 M PBS and then solubilized with dimethyl sulfoxide (DMSO). The absorbance at 490 nm was measured by an automatic enzyme-linked immunosorbent assay reader (Titertek, Helsinki, Finland). Results, plotted in the graph, were generated from the average value of three independent experiments.

### 2.5. Flow Cytometry

5 × 10^5^ mechanical stress untreated and treated SCAPs, serum-starved for 24 h, were collected after being washed in cold PBS twice and centrifuged at 1000 rpm for 3 min. Then cells were fixed with 70% ice-cold ethanol at 4°C for 1 h. DNA content was measured by FAC-Scan flow cytometer (BD Biosciences, San Jose, CA). Cell cycle fractions (G0/G1, S, and G2/M phases) were determined by FCM. The experiment was repeated in triplicate.

### 2.6. Alkaline Phosphatase (ALP) Activity and Alizarin Red Staining

ALP activity was used to evaluate osteogenic differentiation of both treated and untreated SCAPs, assessed with an ALP kit (Sigma-Aldrich). Cells were seeded into 24-well plates (Costar), at a density of 1 × 10^4^ cells/well, after mechanical stress treatment. At the same time, another duplicate plate was applied to measure the protein concentration. Alizarin red staining was carried out, after 21-day culture, using a scanner and destained with cetylpyridinium chloride (CPC) assay. The final data were normalized with the total protein concentration as described above. The experiment was repeated in triplicate.

In this study, ALP analyses were taken to examine the effects of different magnitudes of stress (0 g, 200 g, 250 g, and 300 g) at different time points (6 h, 12 h, 24 h, and 48 h) on SCAPs.

### 2.7. Real-Time Reverse Transcription-Polymerase Chain Reaction (Real-Time RT-PCR)

Total cellular RNA was extracted by using TRlzol reagent (Invitrogen, Carlsbad, CA). Isolated RNA was reversely transcribed using SuperScript III cDNA Synthesis Kit (Invitrogen) according to the manufacturer's instruction. The whole process was at the advantage of diethylpyrocarbonate (DEPC) treated water (Ambion Inc., Austin, USA). RT-PCR was performed using SYBR Premix Ex Taq kit (Takara, Bio, Otsu, Japan) and ABI 7300 real-time PCR system. All primer sets used for the detection of* RUNX2*,* DSPP*,* OSX*, and* GAPDH* ware listed as follows: OSX, Forward 5′-CCT CCT CAG CTC ACC TTC TC-3′ and Reverse 5′-GTT GGG AGC CCA AAT AGA AA-3′;* OCN*, Forward 5′-AGC AAA GGT GCA GCC TTT GT-3′ and Reverse 5′-GCG CCT GGG TCT CTT CAC T-3′;* RUNX2*, Forward 5′-TCT TAG AAC AAA TTC TGC CCT TT-3′ and Reveres 5′-TGC TTT GGT CTT GAA ATC ACA-3′;* DSPP*, forward 5′-ATA TTG AGG GCT GGA ATG GGG A-3′ and Reverse 5′-TTT GTG GCT CCA GCA TTG TCA-3′;* GAPDH*, Forward 5′-GAA GGT GAA GGT CGG AGT C-3′ and Reverse 5′-GAG ATG GTG ATG GGA TTT C-3′. Real-time RT-PCR reaction conditions were 95°C for 30 s, followed by 40 cycles of 95°C for 5 s and 60°C for 31 s.* GAPDH* was used as an internal control to normalize the gene expressions. The data were generated from three independent experiments.

### 2.8. Western Blot Analysis

Mechanical stress-untreated and treated SCAPs were extracted (74 h after mechanical stimuli) and collected after being washed twice with cold PBS and lysed in RIPA lysis buffer (Beyotime, China) containing 1 mM phenylmethylsulfonyl fluoride (PMSF). Cell debris was eliminated by centrifugation at 12,000 rpm for 10 min at 4°C. Protein concentrations were determined via Bio-Rad protein assay kit (Pierce, Rockford, IL). Equal amounts of the protein extracts (30 *μ*g/sample) were separated by 10% SDS-PAGE and blotted onto PVDF membranes (Millipore Co. Bedford, MA, USA) at 300 mA for 1 h in a blotting apparatus (Bio-RAD, CA, USA). Membranes were blocked for 2 h at room temperature with blocking solution (5% w/v skim milk, 0.01 mol/L PBS, 0.1% Tween 20) and subsequently incubated overnight at 4°C. The blots were probed with primary polyclonal antibodies against RUNX2 (1 : 3000; BOSTER, China), DSP (1 : 500; Santa Cruz), OSX (1 : 300; BOSTER, China), and monoclonal antibody against *β*-ACTIN (1 : 1000; ABGENT, Flander, Count, CA, USA). After being washed with PBST (0.1% Tween 20 in 0.01 mol/L PBS), the membranes were incubated with appropriate horseradish peroxidase conjugated secondary antibodies at 1 : 10,000, (Boster Biotech. Co. Ltd., Wuhan, China) at room temperature (22°C) for additional 1 h, developed with enhanced chemiluminescence (ECL) (Santa Cruz Biotechnology) and exposed to X-ray film (Eastman Kodak, Rochester, NY, USA). *β*-ACTIN served as the internal control in these experiments. This experiment was repeated in triplicate.

As to the evaluation of MAPK pathway, SCAPs were firstly serum-starved for 48 h and then treated with 10 *μ*m U0126 (inhibitor of ERK) or/and 10 *μ*m SP60015 (inhibitor of JNK) 1 h before exposure to mechanical force. The cellular proliferation and protein expression were then determined via MTT and ALP assay, respectively [16]. After 0, 15, 30, 60, and 90 minutes of mechanical stress, cellular proteins of MAPKs and phosphorylated MAPKs were instantly detected. Moreover, c-Fos and c-Jun, the downstream of ERK 1/2 and JNK, were examined after 15 minutes of mechanical loading. The data were obtained from at least three independent experiments and quantified by densitometry after normalizing the bands to *β*-actin.

### 2.9. Statistics

The quantitative results were expressed as mean ± SD from three independent experiments performed in triplicate. Independent samples *t*-test and Chi-square test were performed with SPSS Windows v.12.0 software. *P* values less than 0.05 were considered to be statistically significant.

## 3. Results

### 3.1. Mechanical Stress Inhibits the Proliferation of SCAPs

SCAPs were isolated and purified by STRO-1 antibody (Figure 1(b)). The proliferation potential of mechanical stress-treated SCAPs was assessed through the growth curve and flow cytometry.  Figure 1(c) described the effects of 200 g mechanical stress on the proliferation of SCAPs at different time points. Application of mechanical stress resulted in the downregulated proliferation of SCAPs. Such inhibitions were statistically significant at days 5, 7, and 9 (*P* < 0.01). Figures 1(d) and 1(e) described the flow cytometry that 51.92% SCAPs (Figure 1(d)) resided in G_0_G_1_ phases; and the percentage of G_0_G_1_ phases (54.99%, Figure 1(e)) was significantly elevated after mechanical stress treatment. The proliferation index (PI = S% + G_2_M%) in cyclic mechanical group (45.01%) is significantly lower than that in control group (48.08%, *P* < 0.01).

**Figure 2:**
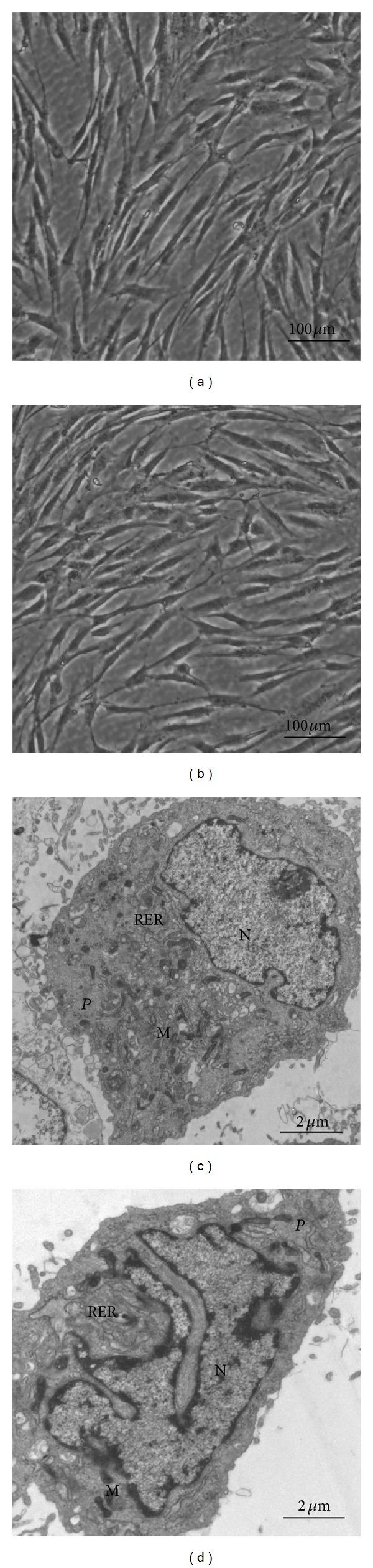
Effects of mechanical stress on the morphology of SCAPs* in vitro.* (a) Untreated SCAPs at day 7. (b) Mechanical stress-treated SCAPs at day 7. (c) TEM analysis showed that untreated SCAPs at day 7 presented the typical ultrastructural features of stem cells, that is, higher nuclear-plasma ratio (N/P ratio) and immature cytoplasmic organelles. (d) Mechanical stress-treated SCAPs at day 7 contained less organelles in the cytoplasm including the rough endoplasmic reticulum (RER) and mitochondria (M). (a), (b) Scale bars = 100 **μ**m; (c), (d) Scale bars = 2 **μ**m.

When the cells were subjected to 200 g mechanical stress for 7 days, the cells showed no particular changes in their morphology and orientation (Figures 2(a) and 2(b)). TEM analysis illustrated that SCAPs possessed the poorly developed cytoplasmic organelles and a high nucleus/cytoplasm ratio that were both the typical ultrastructural features of stem cells (Figure 2(c)). However, after 7-day mechanical stress treatment, organelles (including the rough endoplasmic reticulum and mitochondria) declined and nucleus/cytoplasm ratio became lower (Figure 2(d)).

### 3.2. Mechanical Stress Promotes the Odonto/Osteogenic Differentiation Potential of SCAPs

**Figure 3:**
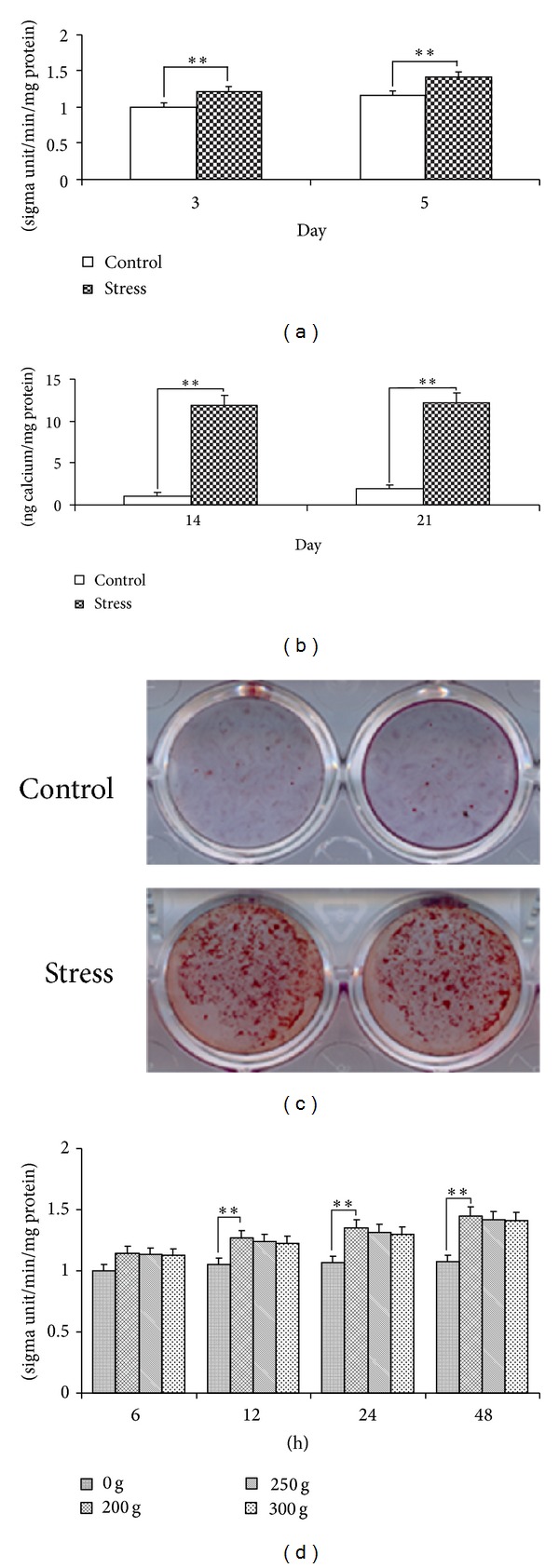
ALP assay and alizarin red staining of SCAPs. (a) Mechanical stress group presented a higher level (*P* < 0.01) of ALP activity than control group after 3-day and 5-day cultures, respectively. (b) After 14-day and 21-day cultures, calcium contents in mechanical stress group were significantly elevated (*P* < 0.01) as compared with those in control group. Values are described as the means ± SD, *n* = 6. (c) After 21-day culture, alizarin red staining demonstrated that mechanical stress group generated more calcification nodules than control group. (d) ALP analyses were performed to test the effects of different magnitudes of stress (0 g, 200 g, 250 g. and 300 g) at different time points (6 h, 12 h, 24 h, and 48 h) on SCAPs. Values were described as the means ± SD, *n* = 3, **P < 0.01.

After 3-day and 5-day mechanical stress treatments, significantly increased ALP activity (*P* < 0.01) was detected (Figure 3(a)). CPC assay showed that the density of calcium concentrations was much stronger in stretched SCAPs at day 14 and day 21 than that of control cells, which also suggested the significantly increased ALP activity at mechanical stress group (*P* < 0.01, Figure 3(b)). Alizarin red staining presented that noticeable mineral nodules were detected in mechanical stress-treated SCAPs at day 21, compared with control group (Figure 3(c)). Besides, we have supplemented the experiments of RT-PCR for adipogenic and chondrogenic differentiation potential of SCAPs, and no significant difference was detected.

**Figure 4:**
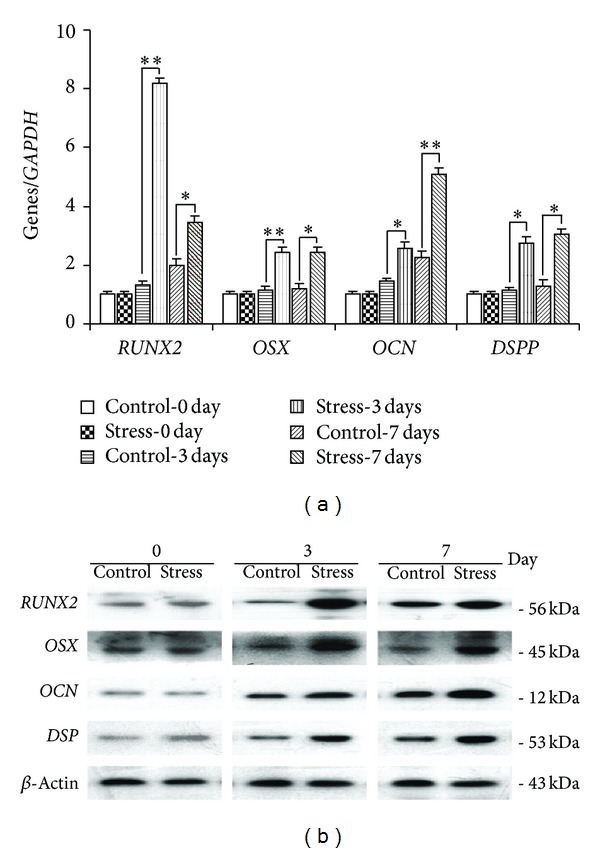
Real-time reverse transcription-polymerase chain reaction and Western blot analyses of SCAPs in different groups. (a) Gene expressions of* RUNX2*,* OSX*,* OCN*, and* DSPP* after 0-day, 3-day, and 7-day cultures.* GAPDH* was used as an internal control for each group. Gene expression was described as a fold change relative to the control group. Values are described as the mean ± SD, *n* = 3. (**2^−ΔΔCt^ ≥ 2, *P* < 0.01; *1 < 2^−ΔΔCt^ < 2, *P* < 0.01). (b) Protein expressions of RUNX2, OSX, and OCN after 0-day, 3-day, and 7-day cultures. *β*-actin was used as a control for each group.

Transcripts of* RUNX2*,* OSX*,* OCN*, and* DSPP* were all detected by real-time PCR and Western blot after mechanical stimulation at day 3 and day 7. All of them experienced an up-going trend. The peak expressions of* RUNX2* and* OSX* were at day 3, while those of the other two were at day 7 (Figure 4).

### 3.3. MAPK Pathway Regulates the Proliferation and Odonto/Osteogenic Differentiation of SCAPs

**Figure 5:**
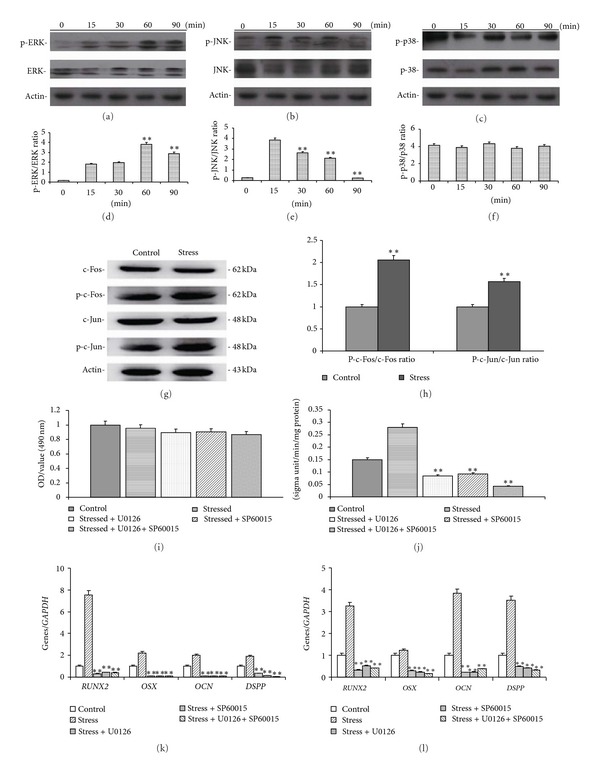
Effects of mechanical stress on MAPK pathway of SCAPs. (a) Protein expressions of phosphor-ERK 1/2 and ERK 1/2 by Western blot. (b) Protein expressions of phosphor-JNK and JNK. (c) Protein expressions of phosphor-p38 and p38. (d) The ratio of pERK/ERK intensity in (a). (e) The ratio of pJNK/JNK intensity in (b). (f) The ratio of pP-38/P38 intensity in (c). (g) Protein expressions of c-Fos and c-Jun by Western blot. (h) The ratio of p-c-Fos/c-Fos and p-c-Jun/c-Jun intensity in (g). (i) MTT analysis of SCAPs under the treatment of stress and/or inhibitors. (j) ALP analysis SCAPs under the treatment of stress and/or inhibitors. (k), (l) Real-time RT-PCR results of the gene expression (*RUNX2*,* OSX*,* OCN*, and* DSPP*) in inhibitors-treated SCAPs, respectively, at day 3 (k) and day 7 (l). Values are described as the means ± SD, *n* = 3. **P < 0.01.

Mechanical stress can activate ERK 1/2 and JNK pathway in SCAPs. Western blot results demonstrated the protein levels of phosphor-ERK 1/2 and ERK 1/2, phosphor-JNK and JNK, phosphor-p38, and p38 in MAPK pathway (Figures 5(a), 5(b), and 5(c)). From the ratio of intensities (Figures 5(d), 5(e), and 5(f)), the phosphor-ERK 1/2 increased dramatically at 60 min (*P* < 0.01) and the phosphor-JNK increased dramatically at 15 min (*P* < 0.01), but the phosphor-p38 was not significantly affected. The activation of ERK 1/2 and JNK was confirmed by the enhanced expression of c-Jun and c-Fos (Figures 5(g) and 5(h)).

Figures 5(i) and 5(j) demonstrated the MTT and ALP results in SCAPs cultured in the absence or presence of mechanical stress and inhibitors. All inhibitors used in this study did not significantly affected cell viability (Figure 5(i)). However, the treatments of stressed SCAPs with U0126 or SP600125 decrease the protein expression (*RUNX2*,* OSX*,* OCN*,* DSPP*; Figures 5(k) and 5(l)), suggesting that the suppression of ERK 1/2 and JNK MAPK pathways can inhibit the stress-induced expression of osteo/odontogenic genes.

## 4. Discussion

It is widely accepted that mechanical loading on cells depends on the magnitude of stress, duration of cyclic load, and loading frequency; and cells respond to mechanical stimuli through the alterations in morphology, structure, and functionality [17, 18]. However, the effects of cyclic stress on proliferation of stem cells are still controversial. The response of cells to cyclic stress could be strongly dependent on the conditions of cyclic stress and the type of stem cells, in which low levels of mechanical stimuli increased cell numbers, while high physiologic levels of mechanical stimuli suppressed cell proliferation [19, 20]. Kokkinos et al. [21] found that mechanical stress affects the mitogenic potential. According to some newly presented studies, endoplasmic reticulum stress, which is associated with the maintenance of pluripotency and differentiation of stem cells, can be reduced by mechanical stress that may reveal further mechanisms of this phenomenon [22–24].

In a previous study, the cessation of progenitor cell proliferation might mark the onset of tissue-specific development during osteogenesis [25]. Besides, mechanical loading was found to contribute to the regulation of osteoblast differentiation by regulating the levels of some osteoblast-specific transcription factors, both at the mRNA and protein levels [26, 27]. As an ongoing research, we evaluated several osteoblast-related markers (*RUNX2*,* OSX*,* DSP*, and* OCN*) in SCAPs by both Western blot and real-time RT-PCR to explore the mechanism of differentiation. The transcription factors* RUNX2* and* OSX* have been identified as the essential elements for osteogenic differentiation.* RUNX2* is pivotal in the early stage of osteogenic differentiation, while* OSX* plays a key role both at the early and later stages, and* OCN* is a later-stage marker [28, 29]. Therefore, it is possible to state that* RUNX2* and* OSX* are higher at day 3 than at day 7. The upgoing expressions of those markers indicate that mechanical stress is vital not only in the early stage of osteogenesis but also in the later stage of tooth and bone formation. Besides,* DSPP* gene and DSP protein, which are involved in the nuclearation and control of the hydroxyapatite mineral phases during dentin calcification, are the putative differentiation markers of odontoblast lineages [29]. Thus, the upregulation of them suggests the enhanced odontogenic differentiation of SCAPs. These abovementioned results also confirmed that* RUNX2* and* OSX* can regulate some osteogenic or odontogenic gene, and confirmed their importance in both osteogenic and odontogenic differentiation.

MAPKs, among some molecules implicated in the stress-inducible signal transduction cascade, have been recently identified in stem cells [30]. These three MAPKs, ERK 1/2, JNK, and p38 are known to be active in dual phosphorylation of Thr and Tyr, which would then phosphorylate and activate the substrates of MAPKs such as transcription factors ATF-1, Elk-1, and c-Jun [31]. In this study, the phosphorylated JNK and ERK protein responded to mechanical stress, increasing after 15 to 60 min of stimuli, but there was no distinguishable difference in phosphorylated p38 protein between control group and test group under mechanical stress. Some researchers have revealed that ERK1/2 is related to the mechanical stimuli via activation of specific ion channels or focal adhesion kinase as mechanical sensors, involved in stress-induced osteogenic-related gene expression [32], while JNK has been associated with cell differentiation, growth arrest, and apoptosis [33, 34]. Although p38 is essential for different cellular functions and even can respond to stress metabolic pathways, controversial views remain raised recently [31, 35, 36]. However, there seems to be little divergent with ERK1/2 for the progress of differentiation [37]. Given this, we sought to examine the further mechanisms of JNK and ERK1/2 at the transcriptional level. One of the downstream mediators of JNK and ERK1/2 is AP-1 transcription factor, which is composed of c-Fos and c-Jun proteins. By checking the downstream of the signaling pathway, it is confirmed that JNK and ERK1/2 are activated with an upregulation of AP-1 members. Furthermore, a recent research contributes the later decrease of ERK1/2 to U0126, a selective inhibitor, and the subsequent osteogenic events to the blocked induction of* RUNX2* [33]. Consequently, these possible mechanisms of mechanotransduction, the activation of JNK and ERK1/2, convert the mechanical stress into intracellular molecular events, which in turn cause protein synthesis, cell proliferation, cell differentiation, and gene expression [38, 39].

In summary, through the application of mechanical stress to SCAPs, this study demonstrates that SCAPs are capable of detecting, transducing, and responding phenotypically to a biomechanical stimulus, which reflects that mechanical force is a feasible way for orthodontic treatment. The expression of* RUNX2*,* OSX*,* OCN*, and* DSPP* strongly suggests the direct differentiation towards the osteogenic/odontogenic cell lineages. Therefore, this work advances our understanding of the mechanical regulation of SCAPs. However, further studies have to be designed and performed to determine the best mechanical stress regimen with the maximal similarity to* in vivo* conditions, under which the differentiation process happens most, and to determine the mechanism by which specific mechanical signals regulate root regeneration, which may reveal novel regulatory pathways and potential targets for the therapeutic intervention.

## 5. Conclusion

In conclusion, this study has demonstrated that mechanical stress can enhance the odonto/osteogenic differentiation of SCAPs via activation of ERK 1/2 and JNK MAPK signaling pathway. These findings provide us a new insight into the influence of vertical stress on the differentiation of stem cells and potential use of mechanical stimuli in tooth engineering. More studies should be performed to investigate other potential mechanisms involved in the differentiation of stress-treated SCAPs, which may smooth the application of mechanical stress in future clinical practice.
